# Acerola-Derived Photorepair System for Eliminating Ultraviolet-Induced Pyrimidine Dimers in Human Cells

**DOI:** 10.3390/nu17050792

**Published:** 2025-02-25

**Authors:** Mamoru Yanagimachi, Tomohiro Umezu, Masakatsu Takanashi, Yoshiki Murakami, Takahiro Ochiya, Masahiko Kuroda

**Affiliations:** 1 Department of Molecular Pathology, Tokyo Medical University, 6-1-1 Shinjyuku-ku, Tokyo 160-8402, Japan; m-yanagi@tokyo-med.ac.jp (M.Y.); t_umezu@tokyo-med.ac.jp (T.U.); takanashi@azabu-u.ac.jp (M.T.); yoshikim@tokyo-med.ac.jp (Y.M.); 2 Department of Life and Environmental Studies, Azabu University, 17-71 Fuchinobe, Chuo-ku, Sagamihara 252-5201, Kanagawa, Japan; 3 Department of Dentistry, Asahi University, Hozumi, Mizuho 501-0296, Gifu, Japan; 4 Department of Molecular and Cellular Medicine, Institute of Medical Science, Tokyo Medical University, 6-7-1 Nishi-Shinjyuku, Shinjyuku-ku, Tokyo 160-0023, Japan; tochiya@tokyo-med.ac.jp

**Keywords:** acerola, extracellular vesicles, photolyase, photorepair, ultraviolet damage

## Abstract

**Background/Objectives**: Ultraviolet B (UV-B) is a significant risk factor for skin damage, as it induces cyclobutane pyrimidine dimers (CPD), which suppress DNA replication and transcription. Photolyase (PHR) is a blue light-dependent enzyme that repairs DNA damage caused by UV irradiation. While it is absent in human, it plays a crucial role in repairing CPD in other organisms. Acerola (*Malpighia emarginata DC*), a fruit with high antioxidant content, is widely consumed for health benefits. This study aimed to identify a novel PHR in acerola and evaluate its photorepair activity. **Methods**: Using RNA-seq data, we cloned the full-length sequence of the acerola PHR gene and constructed an expression vector. A stable transfected HEK293 cell line (HEK293/acPHR) was established. CPD repair activity was analyzed under blue light in these cells, as well as in normal human dermal fibroblasts (NHDFs) supplemented with extracellular vesicles (EVs) from HEK293/acPHR cells and extracellular vesicle-like nanoparticles derived from acerola extract. **Results**: Blue light-dependent CPD reduction was observed in HEK293/acPHR cells compared to control cells following UV-B irradiation. Additionally, CPD repair activity was demonstrated in NHDFs and HEK293 cells treated with EVs from HEK293/acPHR cells and nanoparticles from acerola extract. **Conclusions**: Acerola-derived PHR exhibits the potential to repair UV-induced DNA damage in human cells. Furthermore, EV-mediated delivery of PHR provides a promising avenue for extending photorepair capabilities to other cells. These findings highlight the potential applications of acerola PHR in the prevention and treatment of UV-induced skin damage and related conditions.

## 1. Introduction

Photolyases (PHRs) are members of the cryptochrome/PHR protein family that utilize blue light as an energy source to facilitate the repair of ultraviolet (UV)-induced DNA damage, including cyclobutane pyrimidine dimers (CPD) and pyrimidine-pyrimidone photoproducts (6-4PP) [1,2,3]. Photorepair is the mechanism through which PHRs repair DNA damage. These enzymes are found in various organisms, including photosynthetic bacteria, fungi, plants, fish, birds, and some marsupials [4,5,6,7]. In placental mammals, including humans, PHRs do not possess the ability for DNA repair [8]. Instead, they function as a cryptochrome, acting as a blue-light receptor involved in the regulation of circadian rhythms. Placental mammals employ a distinct mechanism known as nucleotide excision repair (NER) to rectify UV-induced DNA damage [9]. Insufficient repair of UV-induced DNA damage, particularly due to excessive sun exposure, leads to the accumulation of CPD and 6-4PP in genomic DNA. This accumulation has various consequences that increase the risk of premature aging, actinic keratosis, and skin cancer [10].

Clinical trials are underway to demonstrate the efficacy of PHRs in preventing and repairing sun damage in human skin, with the goal of developing skincare products that can mitigate the effects of photoaging. For example, a sunscreen incorporating liposome-encapsulated PHR from the microalga *Anacystis nidulans* was developed. This sunscreen was able to inhibit CPD formation in skin cells caused by UV irradiation and reduce apoptosis by 93% and 82%, respectively, compared to that by a regular sunscreen with SPF 50 [11]. Consequently, advancements in the immobilization of PHRs on liposomes and nanomaterials facilitated their application in various products, including topical creams and sunscreens, thereby ensuring high stability. However, challenges associated with cost, safety, and quality control still need to be addressed.

We have long been interested in the significant contribution of the tropical fruit acerola to health and hypothesized that acerola may also be a valuable source of PHRs. Acerola (*Malpighia emarginata* DC.) is widely recognized as one of the richest natural sources of ascorbic acid and contains various phytonutrients, such as carotenoids, phenols, flavonoids, and anthocyanins, which contribute to its remarkable antioxidant capacity [12]. Acerola was also noted for its other intriguing biological functions, including skin whitening, anti-aging, and suppression of multidrug resistance. Furthermore, acerola juice exerts a protective effect against metal-induced DNA damage [13,14]; however, the role of PHRs in acerola remains unclear. Great interest persists in the scientific, cosmetic, and medical communities in studying this superfruit. If the PHR-mediated photorepair ability of acerola can be demonstrated, it would further enhance the utility of the fruit in maintaining health.

In this study, we aimed to identify a novel PHR gene in acerola. Additionally, we explored the potential for cell-to-cell transmission of PHRs through extracellular vesicles.

## 2. Materials and Methods

### 2.1. De Novo Assembly by Trinity

Acerola fruit pulp (3 g) with seeds removed and five leaves (2 g) were frozen in liquid nitrogen and ground into a fine powder using a mortar and pestle. The homogenized tissue was dissolved in QIAzol, and total RNA was extracted using an RNeasy Mini Kit (Qiagen GmbH, Hilden, Germany) following the manufacturer’s instructions. RNA from acerola fruits and leaves was used to construct, assemble, and analyze de novo transcriptomes. We cloned and sequenced the cDNA encoding a PHR from acerola. Transcriptome sequences were deposited in the DDB J database and used as a proof-of-concept via the BLAST portal (version 2.14.0) to identify candidate PHR/cryptochrome genes.

### 2.2. cDNA Amplification by 5′- and 3′-RACE

The rapid amplification of cDNA ends (RACE) is a technique used to obtain full-length sequences of RNA transcripts. The SMARTer RACE 5′/3′ kit (Takara Bio Inc., Shiga, Japan) was used to synthesize RACE-Ready cDNA as a gene cloning template. Primers were designed using Primer 3Plus (https://www.primer3plus.com/ (accessed on 11 December 2023)) from a part of the predicted sequence of acerola PHR (acPHR) and were PCR-amplified to obtain the acPHR gene fragment. The primer sets were as follows: F: CCTGTTTGGGTAGCTTCAGAGAAGTTGG, and R: AGCTTCTGTACTTTACGGGCCTCCA. Following the instructions of the SMARTer RACE 5′/3′ kit, we designed 5′-RACE and 3′-RACE specific primers, which are as follows: GSP1 (antisense primer for the 5′-race PCR): GATTACGCCAAGCTTAGCTTCTGTACTTTACGGGCCTCCA, and GSP2 (sence primer for 3′-race PCR): GATTACGCCAAGCTTCCTGTTTGGGTAGCTTCAGAGAAGTTGG. We performed RACE cloning and sequencing using AZENTA (Shanghai, China) to obtain the full-length putative acPHR gene.

### 2.3. Comparison of Amino Acid Sequence

The amino acid sequence of acPHR was predicted from the sequenced DNA data by translating the DNA sequences into their corresponding amino acid sequences. Comparison of amino acid sequences among athPHR1, potPHR1, and the putative acPHR was performed using the PROSITE database (https://prosite.expasy.org/prosite.html).

### 2.4. Cell Culture

HEK293 cells and normal human dermal fibroblasts (NHDF; Lot#20TL266554) were purchased from American Type Culture Collection (ATCC, Manassas, VA, USA) and LONZA (Basel, Switzerland). The cells were maintained in Dulbecco’s modified Eagle’s medium (Gibco, Invitrogen, Carlsbad, CA, USA) supplemented with 10% heat-inactivated fetal bovine serum (HyClone, Thermo Fisher Scientific, Waltham, MA, USA) at 37 °C in a humidified atmosphere containing 5% CO_2_, and the adherent cells were harvested through trypsinization.

### 2.5. Establishment of Cell Lines with Stable Expression of potPHR1 and acPHR

The fragments of the open reading frame sequence of potPHR1 and putative acPHR were purchased from IDT (Coralville, IA, USA) and amplified using PCR using the following primers, 5′-GCGGCCACCATGGACTCCAAAAAGAGGAG and 3′-TTCCCTTAATCTGCAGGGCTGA, and 5′-CCCAAGCTTATGGTGTCCCGGGACCAGGC and 3′-CGGGATCCTCAGGTCGGGTTCTCGTAGC for potPHR1 and acPHR, respectively.

The PCR products were inserted into the pcDNA3.1/Hygro (+) expression vector (Invitrogen) according to the manufacturer’s protocol. The established expression vectors were subjected to DNA sequencing to confirm the correct insertion of the full-length potPHR1 or putative acPHR genes. The expression vectors for potPHR1, acPHR, or the corresponding empty pcDNA3.1, were transfected into HEK293 cells using Lipofectamine 3000 (Invitrogen), according to the standard procedure. Forty-eight hours after transfection, the culture medium was replaced with fresh medium containing hygromycin and the cells were cultured for 2 weeks. Hygromycin-resistant clones were established and designated as HEK293/pcDNA3.1, HEK293/potPHR1, and HEK293/acPHR.

### 2.6. Cell Proliferation and Apoptosis Assay

HEK293/pcDNA3.1, HEK293/potPHR1, HEK293/acPHR, and NHDF were seeded at a density of 0.5 × 10^4^ cells/well in 96-well plates (n = 6). NHDF was treated with or without 1.5 × 10^7^ particle/well of concentrated acerola extract (CAE; For details regarding the preparation method of CAE used in this study, please refer to the following CAE section in Section 2. Cell proliferation was assessed at 24 or 72 h of treatment using CellTiter-Glo (Promega, Madison, WI, USA), according to the manufacturer’s protocol. For cell proliferation quantification, a calibration curve was generated using a known number of control cells, and luminescence data from CellTiter-Glo were converted into cell numbers. Background subtraction was performed using cell-free wells.

NHDF was also analyzed for apoptosis induction by similar condition to those of cell proliferation assay using Caspase-Glo 3/7 reagent (Promega). After 24 or 72 h of treatment, the reagent was added to the cells. The luminescence of each sample was determined using a GloMax Multi microplate reader (Promega) according to the manufacturer’s protocol. Each experiment was repeated three times, and data were represented as the mean ± SEM.

### 2.7. Assay for mRNA Expression Using qRT-PCR

The acPHR and β-Actin (ACTB) expression levels were determined using qRT-PCR. Total RNA was extracted from sub-confluent HEK293/pcDNA3.1, HEK293/potPHR1, and HEK293/acPHR cells using an RNeasy MINI Kit (Qiagen). The RNA concentration was determined using a NanoDrop 1000 spectrophotometer (Thermo Fisher Scientific). The expression levels of potPHR1, acPHR, and ACTB were determined by qRT-PCR using the SYBR Green qPCR Master Mix (Thermo Fisher Scientific), assay-on-demand primers, and TaqMan Universal PCR Master Mix Reagent (Applied Biosystems). For acPHR and potPHR1, the following primers were used: F: 5′ GTGGACAAGAGGACCGGAAG 3′, R: 5′ TCTGCTTTCCGCTTCTTTCCT 3′, and F: 5′ GAGGGAGCTGGCAGACAATT 3′, R: 5′ TTCCTTCCTTCACCGTCTGC 3′, respectively. β-Actin (Hs01060665_g1) was used as normalization control. qRT-PCR was performed on a 7900HT thermal cycler (Applied Biosystems, Foster City, CA, USA). The experiment was performed in triplicate, and data are represented as the mean ± SEM.

### 2.8. Extraction of Extracellular Vesicle (EV) Fraction from Cell Culture Supernatant

HEK293/pcDNA3.1, HEK293/potPHR1, and HEK293/acPHR cells were grown in a 100 mm dish. Once they reached confluence (almost all cells reached 1 × 10^7^ cells/dish), the culture medium was replaced with fresh medium (10 mL), and the cells were harvested 24 h later. According to the standard procedure of the ExoEasy Maxi kit (Qiagen), 10 mL of cell culture supernatant was filtered through a 0.45 µm PVDF filter (Millipore, Billerica, MA, USA) to remove cell debris. Equal volumes of the XBP buffer were added and mixed thoroughly. The sample was transferred to an ExoEasy spin column, to which 800 µL of Buffer XE was added, and then centrifuged at 5000× *g* for 5 min. The flow-through was concentrated using 100,000× *g* ultracentrifugation using a TLA-110 rotor 1 (Beckman Colter, Brea, CA, USA) for 70 min at 4 °C to change the elution buffer XE to 30 µL of phosphate-buffered saline (PBS).

### 2.9. Photorepair Activity Measurement

The photorepair activity was measured as previously described [15], with minor modifications. Briefly, sub-confluent cells in 6-well plates were exposed to 312 nm UV-B irradiation (5 mJ/cm^2^) using a UVP Crosslinker CL-1000 (Analytik JenaUS, Upland, CA, USA), followed by 500 nm blue light exposure for 40 min using an LED array system (Amuza, San Diego, CA, USA) after changing to fresh culture medium.

Genomic DNA was extracted using a DNeasy Blood & Tissue kit (Qiagen), and CPD content was measured using an OxiSelect UV-induced DNA Damage ELISA kit (Cosmo Bio, Tokyo, Japan), according to the manufacturer’s protocol (n = 6). Each experiment was repeated three times, and data are represented as the mean ± SEM.

### 2.10. Evaluation of Cell-to-Cell Transmission

Sub-confluent HEK293 cells in 6-well plates were treated with EV fractions from HEK293/pcDNA3.1, HEK293/potPHR1, or HEK293/acPHR cells for 3 h. The number of exogenous particles in each culture was 6 × 10^6^. After replacing the medium with fresh medium, a CPD assay was performed. Each experiment was repeated three times, and data were represented as the mean ± SEM.

### 2.11. CAE

All plant experiments and collection of plant materials complied with the relevant institutional, national, and international guidelines and laws.

CAE was gifted from Nichirei Foods Inc. (Tokyo, Japan). Briefly, juice extracted from uniformly ripe acerola fruits obtained via organic cultivation on a Brazilian farm owned by Nichirei Foods, Inc. was subjected to ultrafiltration using a 100 K molecular weight cut-off filter.

The workflow is as follows: the fruit was rubbed against a stainless-steel mesh to separate the juice. The juice was centrifuged at 5150× *g* for 30 min at 4 °C, and the supernatant was coarsely filtered using glass filter paper (GC-90, GA-100, Advantech Toyo, Tokyo, Japan). The acerola juice was prepared by microfiltration through a cartridge filter (Polysep II Cartridge Filter, 10 in. Code 7, 1.0/0.5 μm, Merck). Subsequently, the juice was concentrated with a 100 K ultrafiltration membrane (Sartocon Slice Hydrosart Cassette 0.1 m2 100 K, 3051446801E-SW, Sartorius). After the juice was concentrated to approximately 1 L, 5 L of Dulbecco’s PBS (D5652-50L, Sigma) was added for solvent replacement and re-concentration. This process was repeated twice. The concentrate was finally passed through a 0.45-μm filter (Opticap XL2 Durapore 0.45-μm, KPHLA02FH3, Merck) to obtain the CAE. The protein concentration of CAE was determined using a Qubit Protein Assay Kit (Thermo Fisher Scientific Inc., Wilmington, NC, USA).

### 2.12. Nanoparticle Tracking Analysis (NTA)

NTA was performed according to the manufacturer’s instructions, using a Nanosight LM10 system (Malvern, Herrenberg, Germany) with a blue laser (405 nm). The nanoparticles in the EV fractions from HEK293/pcDNA3.1, HEK293/potPHR1, HEK293/acPHR, and CAE were illuminated by the laser, and their movement under Brownian motion was recorded in 90 s sample videos, which were analyzed using Nanoparticle Tracking Analysis 2.0 analytical software (Malvern).

Briefly, all samples were diluted with PBS to reach a particle concentration suitable for NTA (1 × 10^8^–2.5 × 10^9^ particles/mL). Capture (shutter and gain) and analysis settings were set manually. All settings were kept constant during each experiment. To determine the total nanoparticle concentration and size distribution profile, data were averaged for each sample across video replicates (at least three triplicate videos for each sample) [16].

### 2.13. Transmission Electron Microscopy (TEM)

EV fractions from HEK293/pcDNA3.1, HEK293/potPHR1, and HEK293/acPHR were fixed with 4% paraformaldehyde and 4% glutaraldehyde in 0.1 M phosphate buffer (pH 7.4) at 37 °C and placed in a refrigerator to lower their temperature to 4 °C. The sections were fixed in osmic acid for 1 h. After washing, they were stained with 0.5% uranyl acetate solution, dehydrated in ethanol, washed, and embedded in epoxy resin. They were then sliced with a diamond cutter and observed under a transmission electron microscope (JEM-1200EX; JEOL Ltd., Tokyo, Japan) at an acceleration voltage of 80 kV, according to the standard procedure.

Negative staining was conducted. Briefly, the CAE was dropped onto a 400-mesh carbon-coated grid in the supporting film, and excess water was removed from the specimen using filter paper. Immediately, a staining solution containing heavy metals such as 2% phosphorus tungstic acid was dropped onto the specimen. Water was then removed from the specimen with a filter paper to dry the specimen. The specimen was then observed using TEM.

### 2.14. Photorepair Activity of CAE in NHDF

NHDF were pre-incubated with or without CAE (5 × 10^8^ particles/well) in 6-well plates for 3 h and then exposed to UV-B irradiation at 312 nm (5 mJ/cm^2^) using the UVP Crosslinker CL-1000 to induce DNA damage, followed by blue light (500 nm) for 40 min. Genomic DNA was extracted 24 h after UV-B irradiation, and the CPD levels were measured using ELISA. Each experiment was repeated three times, and data are represented as the mean ± SEM.

### 2.15. COMET Assay

The single-cell gel electrophoresis assay called COMET was performed in NHDF using a commercial kit (OxiSelect™ Comet Assay, Cosmo Bio). NHDF were pre-incubated with or without CAE (5 × 10^8^ particles/well) in 6-well plates for 3 h and then exposed to UV-B irradiation at 312 nm (5 mJ/cm^2^) using the UVP Crosslinker CL-1000 to induce DNA damage, followed by blue light exposure (500 nm) for 40 min. NHDF were removed from the culture dish 24 h after UV-B irradiation and combined with OxiSelect™ Comet Agarose at 1:10 ratio (*v*/*v*), and 75 µL/well were immediately transferred onto the OxiSelect™ Comet Slide. Slides were maintained horizontally and incubated at 4 °C for 30 min. The slides were then immersed in a lysis buffer for 60 min. The lysis buffer was removed, and the slides were immersed in an alkaline solution for 30 min. The slide was transferred to a horizontal electrophoresis chamber, and a voltage of 1 V/cm was applied for 15 min. After electrophoresis, the slides were washed with H_2_O and 70% ethanol and dried for 30 min. Finally, Vista Green DNA dye was added, and the mixture was incubated at room temperature for 15 min. The cells on the slides were observed under a fluorescence microscope using a fluorescein isothiocyanate filter. DNA damage was quantified by measuring the displacement between the genetic material of the head and tail of the comet. Each experiment was repeated three times, and data are represented as the mean ± SEM.

### 2.16. Statistical Analyses

Data are expressed as the mean ± SEM from three independent experiments. The two treatment groups were compared using Student’s *t*-test. Multiple group comparisons were performed using ANOVA. GraphPad Prism version 5c for Macintosh (GraphPad Inc., La Jolla, CA, USA) was used for statistical analyses. The results were considered statistically significant at *p* < 0.05.

## 3. Results

### 3.1. Full-Length Sequencing of the Open Reading Frame of the Putative acPHR Gene

**Figure 1:**
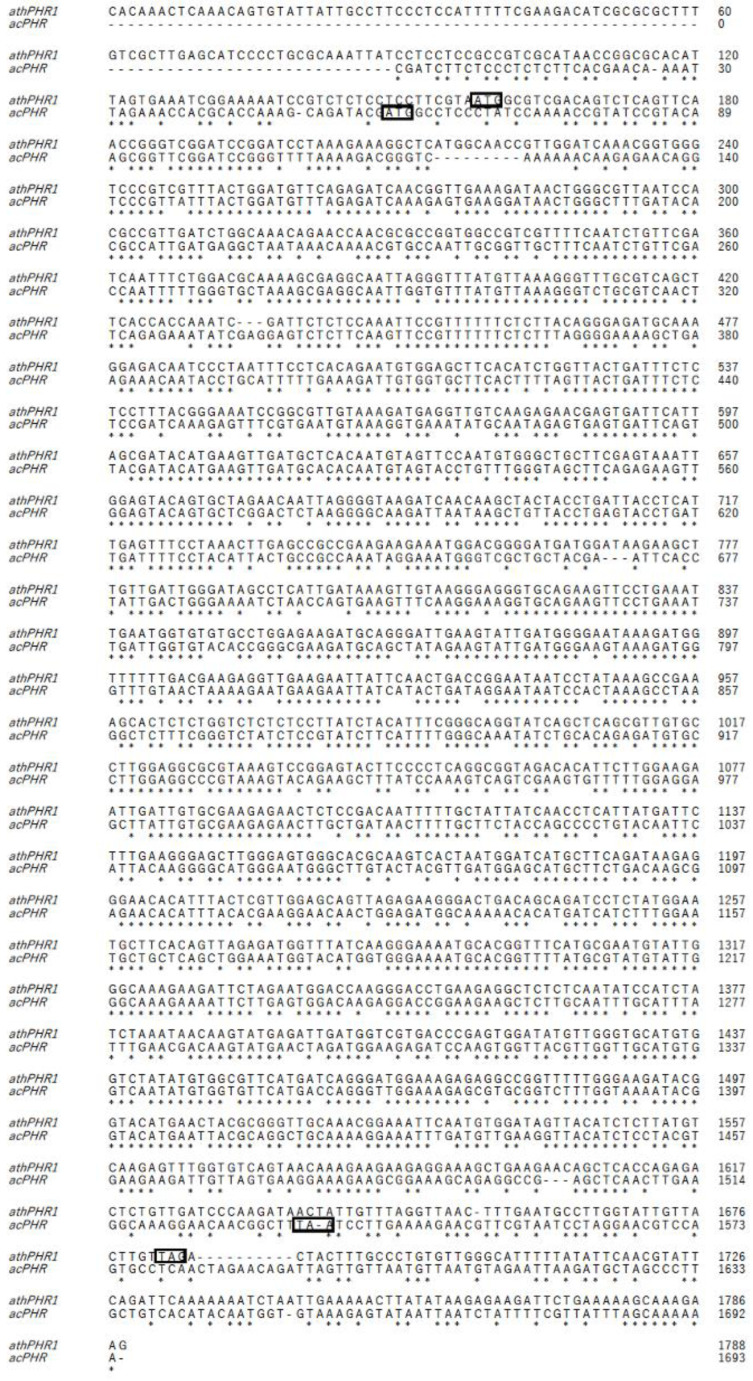
Comparison of coding sequences between *Arabidopsis thaliana* photolyase and putative acerola photolyase genes. Alignment of full-length coding sequences of *Arabidopsis thaliana* photolyase (athPHR1) (**top**) and putative acerola photolyase (acPHR) (**bottom**). Start and stop codons are indicated by boxes.

**Figure 2:**
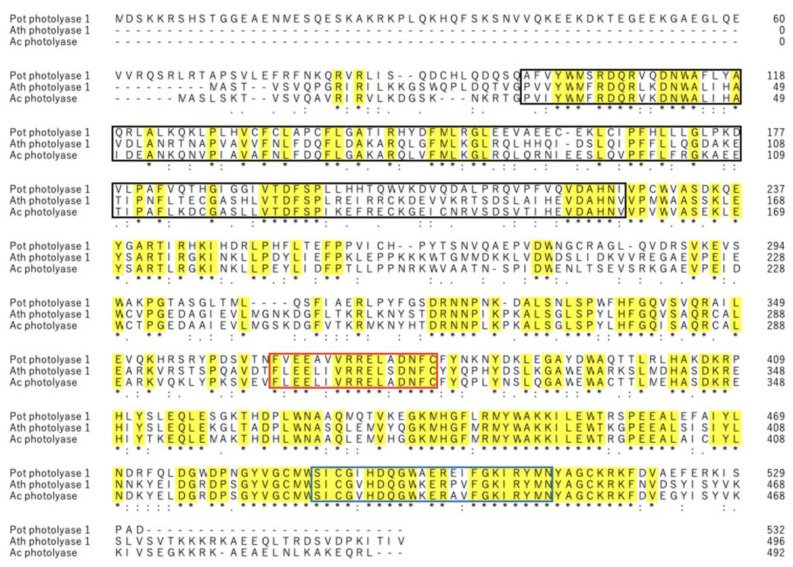
Comparison of amino acid sequences among photolyases from *Potorous tridactylus* (potPHR1), *Arabidopsis thaliana* (athPHR1), and the putative acerola photolyase (acPHR). Alignments of the deduced amino acid sequences from *P. tridactylus* (potPHR1) (**top**), *A. thaliana* (athPHR1) (**middle**), and putative acerola photolyase (acPHR) (**bottom**). Conserved domains, including the photolyase/cryptochrome α/β domain, DNA photolyase class 2 signature 1, and DNA photolyase class 2 signature 2 are highlighted with black, red, and blue boxes, respectively. Yellow indicates identical amino acids in the three photolyases.

Due to the lack of the genetic information regarding the activities of PHR in acerola in any database, to clone acerola PHR (acPHR), we first obtained fastq data from acerola RNA-seq [17] and conducted a reference-guided assembly using Trinity [18] with the *Arabidopsis* PHR (athPHR1) mRNA as a reference. Based on the constructed predicted sequence, the full-length coding sequence of the putative acPHR, from the start codon to the stop codon, was identified using the 5′-RACE and 3′-RACE methods. The full-length cDNA was 1575 bp long and contained an open reading frame of 1479 bp (Figure 1) encoding a peptide of 493 amino acids (Figure 2). The homology between athPHR1 and the putative acPHR was 72.8%.

### 3.2. Putative Amino Acid Sequence

To verify whether the amino acid sequence inferred from the putative acPHR gene sequence contained conserved regions specific to PHR, the amino acid sequences of the putative acPHR were compared with those of athPHR1 (GenBank accession no. NP_001321694), a typical plant PHR, and a porous PHR1 (potPHR1; GenBank accession no. OP753557.1), a typical mammalian PHR. The amino acid sequences between athPHR1 and putative acPHR were 76.7% homologous and those between potPHR1 and putative acPHR were 48.3% homologous. The amino acids conserved in these three PHRs are highlighted in yellow in Figure 2. Referring to the PROSITE database [19], three domains of PHR, namely, cryptochrome α/β domains, DNA PHR class 2 signature 1, and DNA PHR class 2 signature 2, were found in not only the amino acid sequences of athPHR1 and potPHR1 but also in those of putative acPHR. These three domains are highlighted in the black, red, and green boxes in Figure 2, respectively.

### 3.3. Analysis of Photorepair Activity in HEK293/PHR Cell Lines

**Figure 3:**
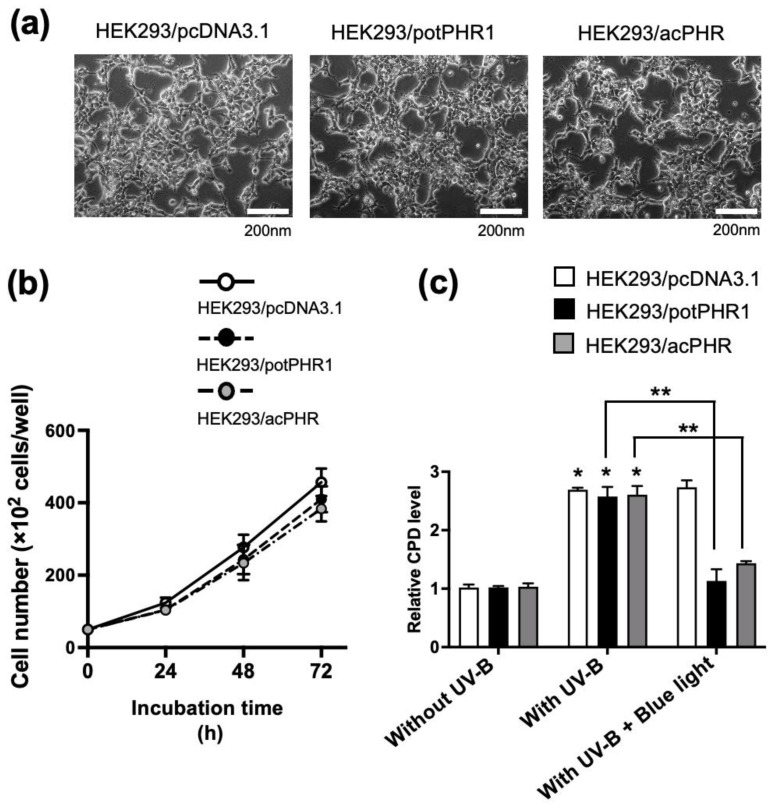
Morphology and photorepair activity in human cells ectopically expressing photolyases. (**a**) Tne representative phase-contrast images of HEK293/pcDNA3.1, HEK293/potPHR1, and HEK293/acPHR cells. Scale bar: 200 nm. (**b**) HEK293/pcDNA3.1 (white dots), HEK293/potPHR1 (grey dots), and HEK293/acPHR (black dots) cells were counted at the indicated time points. (**c**) Quantification of genomic CPD levels in HEK293/potPHR1 and HEK293/acPHR cells following UV irradiation-induced DNA damage and subsequent photorepair by blue light. The vertical axis represents the relative CPD levels normalized to those of the control (HEK293/pcDNA3.1 without UV-B, set as 1 after cell number standardization). White, black, and grey bars indicate HEK293/pcDNA3.1, HEK293/potPHR1, and HEK293/acPHR, respectively. *; *p* < 0.05 compared to that of cells without UV-B exposure in each cell line. **; *p* < 0.05 comparing with that of cells treated with UV-B followed by blue light to those treated with UV-B alone in each cell line.

To examine whether the putative acPHR has blue light-dependent DNA repair activity, we constructed stable transfectant HEK293 cell lines harboring the putative acPHR. pcDNA3.1, which is characterized by hygromycin resistance and a CMV promoter, was selected. Mammalian potPHR1 was selected as the positive control to evaluate cell-based PHR activity in mammalian cells. The empty plasmid pcDNA3.1 or the plasmid containing the potPHR or putative acPHR genes was transfected into HEK293 cells. Hygromycin-resistant clones were established and designated HEK293/pcDNA3.1 (negative control), HEK293/potPHR1 (positive control), and HEK293/acPHR. The representative images of these three cell lines are shown in Figure 3a, indicating no apparent morphological differences among them.

The expression of potPHR1 and the putative acPHR was detected using qRT-PCR in HEK293/potPHR1 and HEK293/acPHR cells, respectively. No expression of potPHR1 or putative acPHR was detected in HEK293/pcDNA3.1 (Table 1). No differences in cell proliferation were observed among HEK293/pcDNA3.1, HEK293/potPHR1, and HEK293/acPHR cells for up to 72 h (Figure 3b).

To investigate the photorepair activity of the putative acPHR, which was ectopically expressed in human cells, HEK293/acPHR cells were exposed to UV-B irradiation (5 mJ/cm^2^), followed by blue light (500 nm) exposure for 40 min. Genomic DNA was extracted, and the CPD levels were measured using a commercially available ELISA kit. UV-B irradiation increased intracellular CPD levels in HEK293/pcDNA3.1, HEK293/potPHR1, and HEK293/acPHR cells. In HEK293/potPHR and HEK293/acPHR cells, blue light exposure reduced CPD levels as much as that observed without UV-B treatment (Figure 3c).

**Figure 4:**
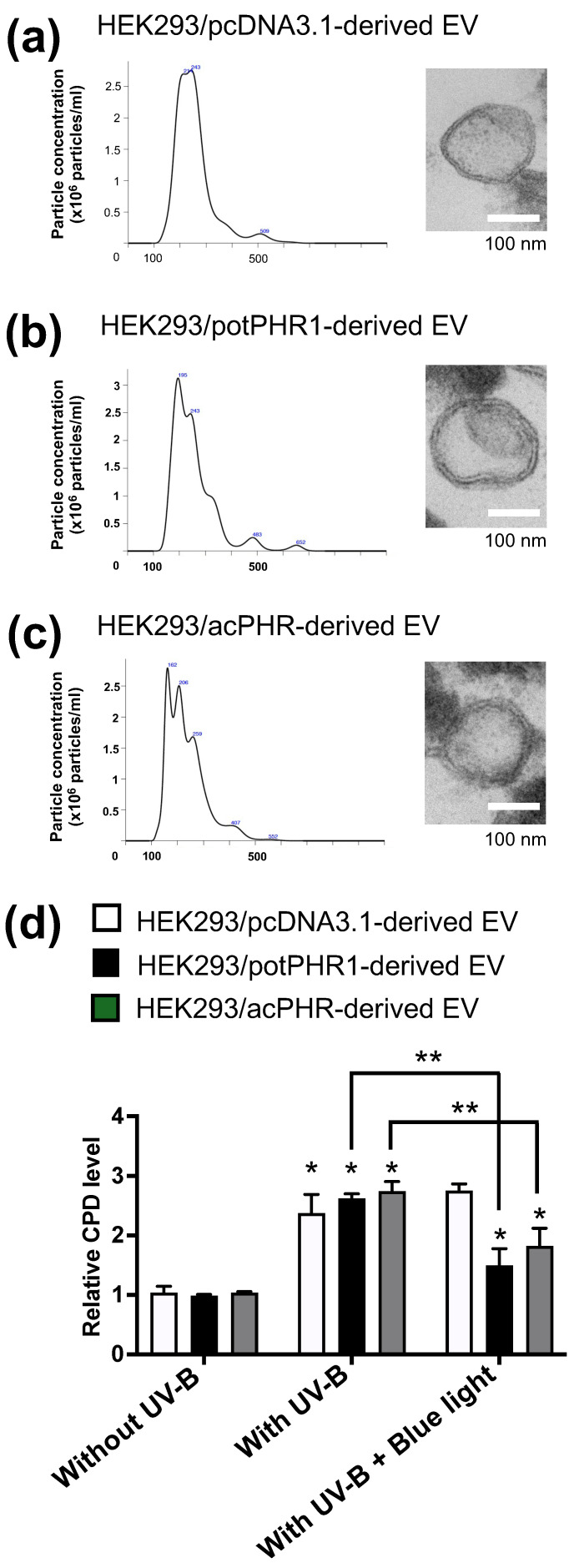
Analysis of the photorepair activity in EVs derived from human cells ectopically expressing photolyase. (**a**) Size distribution profile in NTA (left) and electron micrograph (right) of the EVs isolated from culture supernatants from HEK293/pcDNA3.1 cells. Scale bar: 100 nm. (**b**) Size distribution profile in NTA (left) and electron micrograph (right) of the EVs isolated from culture supernatants from HEK293/potPHR1 cells. Scale bar: 100 nm. (**c**) Size distribution profile in NTA (left) and electron micrograph (right) of the EVs isolated from culture supernatants from HEK293/acPHR cells. Scale bar: 100 nm. (**d**) Quantification of the genomic CPD levels in HEK293 cells treated with HEK293/pcDNA3.1-derived EVs (white), HEK293/potPHR1-derived EVs (black), or HEK293/acPHR-derived EVs (grey) followed by UV irradiation-induced DNA damage and subsequent photorepair by blue light. The vertical axis indicates the relative CPD levels normalized to those of the control (HEK293 cells treated with HEK293/pcDNA3.1-derived EV, without UV-B), set as 1 after standardizing for cell number. *; *p* < 0.05 compared with that of cells without UV-B exposure in each treatment group. **: *p* < 0.05 comparing with that of cells treated with UV-B followed by blue light to those treated with UV-B alone in each treatment group.

To examine the possibility of cell-to-cell transmission of PHR via EVs, we first collected EV from the conditioned media of HEK293/pcDNA3.1, HEK293/potPHR1, and HEK293/acPHR and checked the concentration of particles and physicochemical characteristics, such as particle size, distribution, and shape by NTA and electron microscopy, respectively. The diameters of the major EV fractions from HEK293/pcDNA3.1, HEK293/potPHR1, and HEK293/acPHR cells were approximately 215, 193, and 206 nm, respectively (Figure 4a–c; left panels). Electron microscopy analysis suggested a lipid bilayer-like structure in the EVs released from all three cell lines (Figure 4a–c; right panels).

To confirm whether EVs possessed photorepair activity, EVs from each cell line were added to intact HEK293 cells and incubated for 3 h, followed by exposure to UV-B (5 mJ/cm^2^) and blue light (500 nm) for 40 min. Genomic DNA was then extracted, and CPD content was measured using ELISA. Upon UV-B treatment, increased intracellular CPD levels were observed in cells treated with either type of EV compared to those without UV-B treatment. Cellular CPD levels were significantly reduced by blue light exposure in HEK293 cells incubated with EVs from HEK293/potPHR1 or HEK293/acPHR cells, but not those from HEK293/pcDNA3.1 cells (Figure 4d).

### 3.4. Photorepair Activity in CAE

To investigate whether photorepair activity exists in the EVs from acerola juice, CAE was prepared as an EV fraction and subsequently tested on NHDF. CAE was prepared by Nichirei Foods, Inc. according to the procedure described in the Materials and Methods, and was gifted to us. Approximately 8 mL CAE was prepared from 20 acerolas. CAE is a slightly cloudy, yellow-green liquid with a protein concentration of 6.5 mg/mL. The CAE was divided into small portions and stored in a freezer at −20 °C until use.

**Figure 5:**
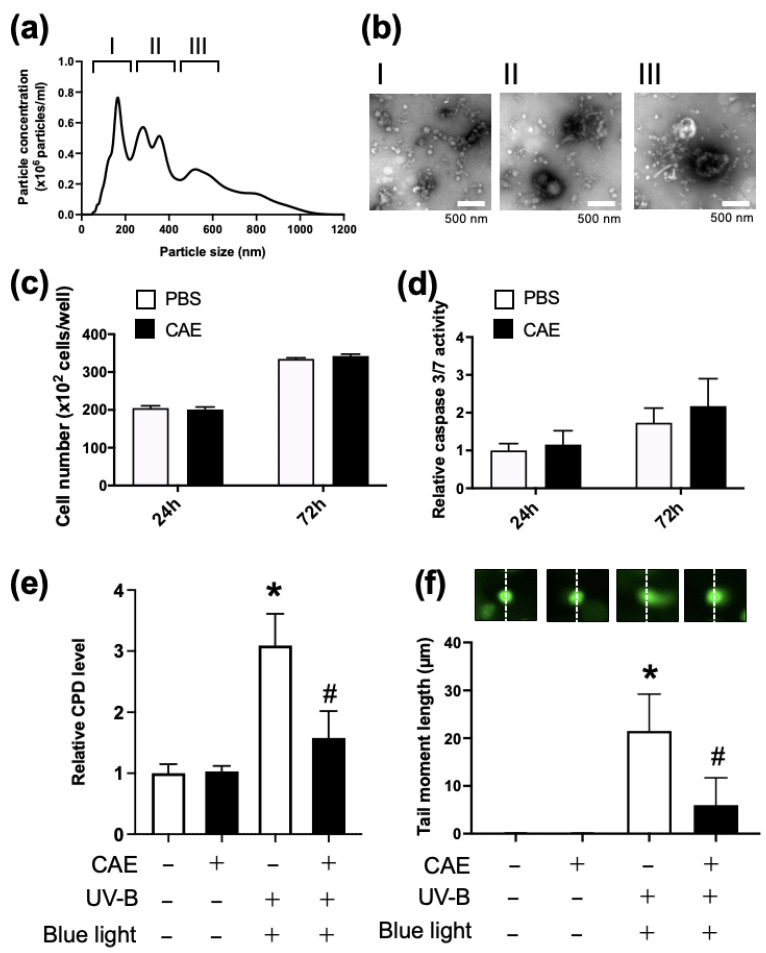
Analysis of photorepair activity in concentrated acerola extract (CAE). (**a**) Size distribution profile of CAE in NTA. Three groups categorized by particle size: I (50–200 nm), II (200–400 nm), and III (400–600 nm). (**b**) Electron micrograph (negative staining) of the representative EVs in each size group of CAE. (**c**) NHDF cell number 24 and 72 h after the addition of CAE to NHDF. The vertical axis represents the number of cells per well. White and black bars indicate NHDF treated with PBS and CAE, respectively. (**d**) Cellular caspase 3/7 levels 24 and 72 h after the addition of CAE to NHDF. The vertical axis shows relative caspase 3/7 levels normalized to levels in control cells treated with PBS, set as 1 after standardization by cell number. White and black bars indicate NHDF treated with PBS and CAE, respectively. (**e**) Photorepair activity in CAE. NHDF were incubated with (black) or without CAE (white) for 3 h and then exposed to UV-B irradiation (5 mJ/cm2) followed by blue light exposure (500 nm) for 40 min. Genomic DNA was extracted 24 h after UV-B irradiation, and CPD levels were measured using ELISA. The vertical axis shows the relative CPD levels normalized to that of the control cells (NHDF without CAE, UV-B, and blue light), set as 1 after standardizing for cell number. *; *p* < 0.05 compared to that of cells without UV-B exposure. #: *p* < 0.05 comparison between the presence and absence of CAE pretreatment. White; absence, black; presence. (**f**) Genotoxicity assessed by COMET assay. The cell culture condition was the same as that in Figure 5e. The COMET assay was performed 24 h after UV-B irradiation. The representative COMET images in each treatment group are shown in upper panels. Dashed line indicates the central position of the nucleus. The tail moment length that defines the distance between the center of mass of the tail and the center of the head of the cell is shown in histogram as mean ± SD (n = 5). *; *p* < 0.05 compared to that of cells without UV-B exposure. #: *p* < 0.05 comparison between the presence and absence of CAE pretreatment. White; absence, black; presence.

NTA showed that the CAE solution contained nanoparticles at a concentration of 1 × 10^11^ particles/mL and consisted of three major fractions with diameters of 50–180 (I), 180–530 (II), and 530–770 nm (III) (Figure 5a). Negative staining in electron microscopy analysis also showed vesicles of various sizes (Figure 5b).

To manage the CAE-dependent adverse events, we first examined the effect of CAE on the proliferation and apoptosis of NHDF. CAE did not affect the proliferation (Figure 5c) or apoptosis (Figure 5d) of NHDF after 24 h and 72 h of incubation.

To evaluate the photorepair activity of CAE, NHDF were pre-incubated with or without CAE for 3 h and then exposed to UV-B irradiation (5 mJ/cm^2^), followed by blue light exposure (500 nm) for 40 min. Genomic DNA was extracted 24 h after UV-B irradiation, and CPD levels were measured using ELISA. This showed that CAE did not affect cellular CPD content when NHDF did not receive UV-B or blue light. There was a 3-fold increase in intracellular CPD after UV-B irradiation in NHDF without CAE treatment. This UV-B-dependent increase in cellular CPD was partially attenuated by CAE and blue light exposure (Figure 5e).

Finally, we performed COMET assay, a single cell gel electrophoresis assay, to evaluate the extent of genomic DNA damage and to assess the effect of CAE and blue light exposure on UV-B-dependent DNA damage in NHDF. Cell culture conditions were the same as those shown in Figure 5e. In this assay, we measured the tail moment length, which is defined as the distance between the center of mass of the tail and the center of the head of the cell. Without UV-B irradiation, the tail moment length could not be observed in NHDF. A tail moment length of 20 µm was observed in NHDF irradiated by UV-B. Incubation with CAE or blue light partially suppressed the UV-B-dependent increase in tail moment length.

## 4. Discussion

To the best of our knowledge, this is the first study identifying a novel PHR from acerola that repairs DNA damaged by UV-B irradiation. The putative mRNA and amino acid sequences of acPHR showed 72.8% and 76.8% homology to those of athPHR1, respectively (Figure 1 and Figure 2). The comparison of the amino acid sequences of potPHR1, a mammalian PHR, and putative acPHR exhibited only 43.8% homology. However, essential catalytic domains of PHRs, such as cryptochrome α/β domains, DNA PHR class 2 signature 1, and DNA PHR class 2 signature 2, were highly conserved among athPHR1, potPHR1, and putative acPHR (Figure 2) [19].

Based on the putative mRNA sequence, HEK293/acPHR cells with stable expression of the putative acPHR gene were established. HEK293/pcDNA3.1 and HEK293/potPHR1 cells were also established using the stable expression of empty vector and vector harboring potPHR1 genes as negative and positive control cell lines, respectively. During the preparation of these cell lines, specific expression of potPHR1 and acPHR mRNA was confirmed in HEK293/potPHR1 and HEK293/acPHR cells (Table 1). No differences in cell morphology and proliferation were observed among the three cell lines, suggesting no toxicity of exogenously transfected PHR genes (Figure 3a,b). The cellular CPD content was increased by UV-B irradiation in these cell lines to a similar extent. In HEK293/potPHR1 and HEK293/acPHR cells, CPD levels were recovered by blue light exposure, but not in HEK293/pcDNA3.1 (Figure 3c). These data suggest that HEK293/acPHR cells possess blue light-dependent photorepair activity derived from the exogenously transfected putative acPHR gene. These results provide strong evidence for the presence of PHR in acerola. Earlier studies detected DNA repair activity in acerola juice [20,21], suggesting that the active form of PHR exists outside the cells in acerola. Therefore, we hypothesized that the acPHR protein is localized in extracellular nanovesicles, such as exosomes. As shown in Figure 4a–c, NTA revealed that the EVs derived from HEK293/pcDNA3.1, HEK293/potPHR1, and HEK293/acPHR had similar size patterns and concentrations. Electron microscopic analysis suggested the existence of typical features of the lipid bilayer in the EVs of these three cell lines. Then, we tested whether these EVs possessed photorepair activity in intact HEK293 cells. Cellular CPD levels were significantly reduced following blue light exposure in HEK293 cells pre-incubated with EVs from HEK293/potPHR1 or HEK293/acPHR but not in cells incubated with EVs from HEK293/pcDNA3.1. (Figure 4d). These data suggest that the PHR protein is localized not only inside cells but also outside cells, such as in EVs, and that EVs can transfer PHR activity to other cells.

However, studies using HEK293 cell-derived cell lines were based on artificial conditions. Thus, we next evaluated the photorepair activity of the acerola extract in NHDF, which seemed to be closer in terms of physiological conditions, to humans than genetically transformed cell lines. Both NTA and electron microscopy showed vesicles of various sizes in the CAE, suggesting that they contained both extracellular and intracellular vesicles (Figure 5a,b). We first evaluated the effect of CAE on cell proliferation and apoptosis in NHDF and found no cellular toxicity of CAE for up to 72 h (Figure 5c,d). Figure 5e shows the increase in cellular CPD by UV-B irradiation and restoration of CPD content by CAE treatment and blue light exposure, which suggests the susceptibility of NHDFs to DNA damage by UB-V irradiation and the possibility of blue light-dependent PHR activity in CAE. To monitor DNA damage induced by UV-B irradiation via another method other than cellular CPD content measurement, we performed COMET assay in NHDF (Figure 5f). The COMET assay is thought to be a general method for monitoring UV-dependent cell damage, involving both thymine dimer formation and double-stranded DNA breaks. CAE and subsequent blue light exposure could restore CPD content by intrinsic PHR activity, although they seemed to fail to recover double-stranded DNA breaks and other DNA damages induced by UV-B. Therefore, CAE only partially recovered the tail length moment in the COMET assay. These findings indicate the presence of PHR in the acerola extract and the transfer of PHR-dependent photorepair activity to target cells by PHR-containing EVs.

Other studies provided preclinical and clinical evidence of reduced UV-induced DNA damage in the skin with the topical application of sunscreens containing exogenous PHR for industrial utility [19]. Photosome-V was developed and used in industrial applications and contains PHR derived from *Anacystis nidulans*, a Cyanobacteria. It has shown protective effects in human skin against CPD increase, DNA damage, inflammation, and stress signals induced by UV-B [22]. From this perspective, PHR-liposomes were thought to be more promising than intact PHR for cosmetic and pharmaceutical preparations. However, these PHR-containing liposomes are difficult to use as commercially available products because of the numerous steps and high cost of factory-based manufacturing with adequate quality control. Contrastingly, preparing acPHR-containing CAE from acerola fruit seems to be easier and less costly than preparing PHR liposomes, for example, from Antarctic microalgae [23]. This advantage is due to the stable supply of acerola from global agricultural markets and the development of a simple method for preparing CAE without special reagents or equipment, as described in the Materials and Methods. Additionally, concerns regarding the safety of acPHR and CAE are low because acerola is widely consumed globally. Thus, acPHR may have powerful cosmetic, industrial, and aesthetic medicinal applications.

## 5. Limitations

This study had three limitations. (1) Preliminary western blotting analysis using commercially available antibodies against athPHR1 failed to detect the putative acPHR protein. Therefore, the molecular weight and the intracellular and extracellular distributions of acPHR remain unknown. (2) Additionally, to better characterize the molecular mechanism of acPHR, including the blue light-dependent enzyme reaction, a specific antibody against acPHR is required. (3) Although there are multiple mechanisms for repairing UV-induced DNA damage in cells, the contribution of exogenous PHR in CAE is unclear. Future studies are needed to compare the contribution of acPHR with that of antioxidants and other mechanisms.

## 6. Conclusions

Conclusively, this is the first study to identify the ability of acerola PHR to repair UV-induced DNA damage in human cells and report that PHR-containing EVs may transfer their photorepair ability to other cells. This study provides novel insights into the functions and potential applications of acerola PHR in the treatment and prevention of UV-induced skin damage and related diseases.

## Figures and Tables

**Table 1:** PHR Gene Expression Analysis.

	Ct Value		
	potPHR1	acPHR	β-actin
HEK293/pcDNA3.1	undetermined	undetermined	18.49 ± 0.12
HEK293/potPHR1	14.05 ± 0.35	undetermined	18.13 ± 0.03
HEK293/acPHR	undetermined	17.36 ± 0.99	18.36 ± 0.1

Gene expression of potPHR1, acPHR, and b-actin in HEK293/pcDNA3.1, HEK293/potPHR1, and HEK293/acPHR cells by qRT-PCR. Experiment was performed in triplicate, and data are represented as mean ± SEM.

## Data Availability

All data generated or analyzed during this study are included in this published article.
